# Muscle Strength as a Key Independent Predictor of Arterial Stiffness and Metabolic Syndrome in Aging Mexicans: Unveiling the Sarcopenic Obesity Paradox

**DOI:** 10.3390/jcm15156006

**Published:** 2026-08-02

**Authors:** Exal Garcia-Carrillo, Paz Pezoa-Fuentes, Eduardo Guzmán-Muñoz, Yeny Concha-Cisternas, Felipe Montalva-Valenzuela, Jorge Olivares-Arancibia, Joaquín González-Aroca, Guillermo Cortés-Roco, Rodrigo Yáñez-Sepúlveda

**Affiliations:** 1 Department of Physical Activity Sciences, Faculty of Education Sciences, Universidad Católica del Maule, Talca 3480112, Chile; paz.pezoa.fu@gmail.com; 2 Department of Physical Activity Sciences, Universidad de Los Lagos, Osorno 5290000, Chile; 3 Escuela de Pedagogía en Educación Física, Facultad de Educación, Universidad Autónoma de Chile, Talca 3460000, Chile; eduardo.guzman@cloud.uautonoma.cl; 4 Escuela de Kinesiología, Facultad de Salud, Universidad Santo Tomás, Talca 3460000, Chile; yenyconchaci@santotomas.cl; 5 Vicerrectoría de Investigación e Innovación, Universidad Arturo Prat, Iquique 1100000, Chile; 6 Escuela de Entrenador en Actividad Física y Deporte, Facultad de Ciencias Humanas, Universidad Bernardo O’Higgins, Santiago 8370040, Chile; felipe.montalva@ubo.cl; 7 AFySE Group, Research in Physical Activity and School Health, School of Physical Education, Faculty of Education, Universidad de las Américas, Santiago 7500975, Chile; jolivares@udla.cl; 8 Escuela de Kinesiología, Facultad de Salud, Universidad Santo Tomás, La Serena 1720236, Chile; jgonzalez180@santotomas.cl; 9 Facultad de Ciencias de la Vida, Magíster en Evaluación y Planificación del Entrenamiento Deportivo, Universidad Viña del Mar, Viña del Mar 2520000, Chile; guillermo.cortes@uvm.cl; 10 Escuela de Ciencias del Deporte, Facultad de Educación y Humanidades, Universidad Andres Bello, Viña del Mar 2200055, Chile; rodrigo.yanez.s@unab.cl; 11 School of Medicine, Universidad Espíritu Santo, Samborondón 092301, Ecuador

**Keywords:** hand strength, pulse pressure, waist circumference, aged, middle aged, sex factors, cardiometabolic risk factors, adiposity, geriatric assessment, body composition

## Abstract

**Background/Objectives**: The cardiometabolic consequences of sarcopenic obesity (SO) remain poorly characterized in Latin America. We hypothesized that SO uniquely concentrates aortic stiffness, metabolic syndrome, and vitamin D deficiency in older Mexican adults, and that handgrip strength exhibits a dose–response relationship with cardiometabolic risk independent of adiposity. **Methods**: We conducted a cross-sectional analysis of 2087 adults ≥ 50 years from the Mexican Health and Aging Study (MHAS) 2012. Four phenotypes were defined using EWGSOP2 criteria: lean–fit, lean–sarcopenic, obese–non-sarcopenic, and obese–sarcopenic (BMI ≥ 30 kg/m^2^). Outcomes were pulse pressure (PP, aortic stiffness proxy), metabolic syndrome (modified IDF criteria), and serum 25(OH)D. Normality tests confirmed non-normal distribution (*p* < 0.001). Given heteroscedasticity (Breusch–Pagan *p* < 0.001), HC3 robust standard errors were used. Regression models were sex-adjusted and sex-stratified. **Results**: The sample showed high cardiometabolic burden: mean HbA1c 6.91% (35.3% ≥ 6.5%); metabolic syndrome 57.6%; mean PP 60.7 ± 17.9 mmHg; mean gait speed 0.708 m/s (70.4% slow). Phenotype distribution: lean–fit 12.3%; lean–sarcopenic 47.3%; obese–non-sarcopenic 8.7%; obese–sarcopenic 31.7%. In multivariable models, handgrip strength was an independent protective predictor of PP (beta = −0.09 per kg; 95% CI: −0.17 to −0.01; *p* = 0.033), with a sex-specific effect in women (beta = −0.13; *p* = 0.010). **Conclusions**: SO is highly prevalent in older Mexican adults and associates with an adverse cardiometabolic profile. Handgrip strength shows a protective dose–response association with cardiometabolic risk independent of adiposity, identifying muscle function as a candidate modifiable factor that warrants confirmation in longitudinal and interventional studies. The obese–non-sarcopenic phenotype exhibited the lowest PP, unveiling the sarcopenic obesity paradox: adiposity without muscle function loss does not promote arterial stiffening.

## 1. Introduction

The global epidemiological transition has contributed to a complex health scenario in low- and middle-income countries, where excessive adiposity accumulation increasingly coexists with a progressive loss of lean mass and muscle function [1,2]. From a biological perspective, excess adiposity is not metabolically inert; the pathological expansion of adipose tissue through hypertrophy and hyperplasia promotes the secretion of adipokines, cytokines, and chemokines with systemic effects on metabolism, inflammation, and organ function [3,4]. This overlap is clinically relevant because it gives rise to sarcopenic obesity (SO), a distinct phenotype characterized by the concurrence of obesity and sarcopenic features, which may impose a higher cardiometabolic burden than either condition alone [5]. However, the mechanisms linking SO to cardiovascular risk remain incompletely understood [6]. Given its combined metabolic and functional components, SO may contribute to cardiovascular risk through vascular alterations driven by chronic inflammation, insulin resistance, and impaired muscle–vascular interactions. Arterial stiffness, a subclinical marker of vascular aging that predicts hypertension, stroke, and mortality [7], may therefore represent a key mechanistic pathway linking SO to adverse cardiovascular outcomes. In this context, pulse pressure (PP), which reflects the reduced compliance of the aorta and large arteries, has been widely used as an indirect marker of arterial stiffness and has been independently associated with adverse cardiovascular outcomes [8]. 

Obesity and sarcopenia may jointly accelerate arterial stiffening through chronic inflammation, oxidative stress, and insulin resistance [6]. Yet, few studies have examined whether muscle strength (i.e., handgrip strength) is independently associated with adiposity, arterial stiffness, and metabolic syndrome. From a mechanistic perspective, low muscle strength could exacerbate the deleterious effects of central obesity on the vasculature, whereas preserved strength might confer resilience despite excess adiposity [9]. This possibility gives rise to what we term the sarcopenic obesity paradox: older adults with obesity but relatively preserved muscle strength may exhibit lower arterial stiffness and metabolic risk than their counterparts with similar adiposity but lower strength. Supporting this concept, Stenholm et al. demonstrated in a 33-year follow-up study that obese individuals with low handgrip strength had the highest mortality risk (HR 1.23), whereas overweight and obese participants with high handgrip strength exhibited significantly lower mortality than normal-weight individuals with high strength [9]. Moreover, muscle strength appears to be a more important predictor of cardiometabolic risk than muscle mass per se [10], and low muscle mass has been directly associated with arterial stiffness [11,12]. Collectively, these findings suggest that preserved muscular fitness may confer resilience against the vascular consequences of excess adiposity.

Mexico provides an instructive context: sarcopenic muscle loss has been documented as early as the fifth decade of life [13], suggesting that the aging Mexican population may be particularly vulnerable to the dual burden of adiposity accumulation and muscle deterioration. Nationally representative data indicate that overweight and obesity affect approximately 75% of adults aged 50 years and older [14], while the metabolic syndrome prevalence exceeds 70% in this age group, one of the highest rates reported globally [15]. Despite this alarming epidemiologic profile, the cardiometabolic consequences of SO remain largely uncharacterized in Latin American populations. Specifically, no prior study has examined whether arterial stiffness, measured through PP, mediates the relationship between central obesity and metabolic syndrome in older Mexicans, nor has the potential modifying role of muscle strength been systematically evaluated. Furthermore, sex differences, particularly the extraordinary excess of metabolic syndrome in postmenopausal women, represent a critical knowledge gap, as women exhibit both higher adiposity and higher arterial stiffness after menopause [16], yet the contribution of muscle strength to this disparity remains unexplored.

Therefore, the present study had three primary objectives: (1) to characterize the distribution of obesity, probable sarcopenia, and their combination (SO) in a nationally representative sample of Mexican adults aged 50 years and older; (2) to examine whether handgrip strength is independently associated with PP (a surrogate marker of arterial stiffness) after accounting for waist circumference and to formally test its role as a statistical mediator of the waist circumference–PP relationship using bootstrapped indirect effects; and (3) to examine whether these mediation pathways differ between men and women, with particular attention to the postmenopausal female subgroup. We hypothesized that lower handgrip strength would significantly mediate the obesity–arterial stiffness relationship, and that this indirect effect would be stronger in women, consistent with their higher metabolic syndrome burden.

## 2. Materials and Methods

### 2.1. Study Design and Participants

The Mexican Health and Aging Study (MHAS) is a prospective population-based panel study of adults aged ≥ 50 years, with waves in 2001, 2003, and 2012, based on a nationally representative multistage probabilistic sample [17]. In 2012, the National Institute of Public Health (INSP) collected biological samples and physical fitness measurements from 2016 participants [18]. The final analytical sample was *n* = 2087: 1248 women (59.8%) and 839 men (40.2%).

### 2.2. Phenotype Classification and Physical Fitness Variables

Four mutually exclusive phenotypes were defined based on the presence or absence of obesity and probable sarcopenia. Obesity was defined as BMI ≥ 30 kg/m^2^ according to World Health Organization criteria [19]. Although age-adjusted BMI cut-offs (e.g., >27 kg/m^2^) have been proposed for older adults [20], we retained the WHO definition to ensure comparability with the majority of Latin American epidemiological studies and because the mean BMI in our sample (29.2 kg/m^2^) exceeds the adjusted threshold, minimizing misclassification risk. Probable sarcopenia was defined following the revised European Working Group on Sarcopenia in Older People (EWGSOP2) criteria [21] as low handgrip strength (<27 kg for men, <16 kg for women) and/or slow gait speed (<0.8 m/s). Although gait speed is formally used in EWGSOP2 to confirm severity rather than to diagnose probable sarcopenia, we included it as a functional indicator given the absence of muscle mass measurements and its strong prognostic value in older populations. Accordingly, the four phenotypes were: lean–fit (no obesity, no probable sarcopenia); lean–sarcopenic (no obesity, with probable sarcopenia); obese–non-sarcopenic (obesity, no probable sarcopenia); and obese–sarcopenic (obesity and probable sarcopenia).

Handgrip strength was measured using a Baseline hydraulic dynamometer (Fabrication Enterprises Inc., White Plains, NY, USA). Participants were seated with their shoulder adducted and neutrally rotated, elbow flexed at 90 degrees, and forearm in a neutral position. The dynamometer handle was adjusted to the second position to accommodate hand size. Four alternating attempts were performed on each hand (left–right–left–right), and the maximum value across all attempts (regardless of hand) was recorded in kilograms. The device’s “peak-hold” pointer was reset before each attempt to capture the maximum isometric force.

Gait speed was assessed over a 4 m course. Participants were instructed to walk “at their usual pace” from a standing start. The time was measured with a stopwatch, and the test was performed twice. The mean time of the two trials was converted to meters per second (m/s) [22].

Single-leg balance (static balance) was measured barefoot. Participants were instructed to stand on one foot (starting with the right) with their arms relaxed at their sides and eyes open. The test ended when the participant either: (a) lowered the raised foot, (b) moved the standing foot from its original position, (c) opened their eyes (if closed in other versions, but here they are open), or (d) grasped an external object for support. The maximum time recorded was 10 s per foot, with one attempt per foot [23].

### 2.3. Biomarkers and Blood Pressure

Venous blood samples were processed at the INSP central laboratory following MHAS protocols [18]. The following biomarkers were measured: 25-hydroxyvitamin D (25[OH]D) by immunoassay, with deficiency defined as <20 ng/mL; high-density lipoprotein cholesterol (HDL-C) and total cholesterol by enzymatic method; high-sensitivity C-reactive protein (hs-CRP) by immunoturbidimetry; thyroid-stimulating hormone (TSH) by electrochemiluminescence; and HbA1c from capillary blood using the A1CNow point-of-care device (PTS Diagnostics Inc., Whitestown, IN, USA) [18]. Blood pressure was measured twice using an OMRON electronic device (OMRON HEALTHCARE Co., Ltd., Kyoto, Japan) after a 5 min rest period, with participants seated and feet flat on the floor. The mean of the two readings was used for analysis. The two office readings were separated by a median interval of 7 min (interquartile range 5–12). PP was calculated as the systolic blood pressure (SBP) minus the diastolic blood pressure (DBP), with a threshold of ≥60 mmHg indicating elevated arterial stiffness. PP is a surrogate (proxy) marker of aortic stiffness rather than a direct measure such as carotid–femoral pulse wave velocity and is additionally influenced by stroke volume, heart rate, and peripheral resistance beyond arterial compliance [8].

### 2.4. Metabolic Syndrome

Metabolic syndrome was defined according to the modified International Diabetes Federation criteria (IDF) [24], which require central obesity (waist circumference > 94 cm for men, >80 cm for women) plus at least two of the following four components: (1) elevated HbA1c ≥ 5.7% (reflecting impaired fasting glucose); (2) elevated blood pressure (SBP ≥ 130 mmHg or DBP ≥ 85 mmHg) or current antihypertensive treatment; (3) reduced HDL-C (<40 mg/dL in men, <50 mg/dL in women); and (4) elevated triglycerides (≥150 mg/dL) [24,25]. The IDF criteria were selected because they prioritize central obesity as a mandatory component, which aligns with our focus on abdominal adiposity as a key driver of cardiometabolic risk.

### 2.5. Statistical Analysis and Diagnostics

Normality was assessed using Shapiro–Wilk and Kolmogorov–Smirnov tests (non-normal distribution for all variables, *p* < 0.001). Descriptive comparisons between sexes were performed using the Mann–Whitney U test (continuous variables) and chi-square test (dichotomous variables). Comparisons across phenotypes were performed using the Kruskal–Wallis test. Homogeneity of variances was assessed using Levene’s test. Heteroscedasticity was confirmed by the Breusch–Pagan test (BP = 44.4; *p* < 0.001), leading to the use of HC3 robust standard errors in all OLS models [26]. Multicollinearity was assessed for all independent variables included in the regression models using tolerance values and variance inflation factors (VIF). Variables with tolerance values <0.10 and/or VIF values >10.0 were considered indicative of severe multicollinearity and were excluded from the final models. Therefore, the final multivariable models excluded BMI and retained waist circumference as the index of central adiposity (a more sensitive predictor of cardiometabolic risk); BMI was retained exclusively for dichotomous obesity classification (≥30 kg/m^2^) in phenotype definition. After this specification, all VIF values were <5. All multivariable OLS and logistic regression models were adjusted for sex and were replicated stratified by sex (men: *n* = 707; women: *n* = 1052). Regression models were restricted to participants with complete data on all covariables (*n* = 1757; 84.0% of the total analytical sample). Participants excluded from regression models (*n* = 330) did not differ significantly from the included participants in age, sex distribution, or BMI (all *p* > 0.05), suggesting that missing data were unlikely to introduce substantial selection bias. In addition, included and excluded participants did not differ in the core outcome (pulse pressure) or in the principal exposures (handgrip strength, waist circumference, and gait speed), nor in vitamin D or sex (all *p* > 0.29); excluded participants were, however, slightly younger (58.0 vs. 62.5 years; *p* < 0.001); so, the data cannot be assumed to be missing completely at random, and the potential for bias is addressed in the Limitations. Handgrip strength tertiles were defined at the 33rd and 66th percentiles. To characterize the shape of the handgrip strength–PP relationship, we additionally fitted restricted cubic splines (natural cubic splines, three degrees of freedom) for continuous handgrip strength, adjusted for age and sex, and tested departure from linearity with a Wald test on the non-linear spline terms. To evaluate whether handgrip strength statistically mediated the association between adiposity and PP, we conducted a formal mediation analysis (Baron–Kenny framework) of the waist circumference → handgrip strength → PP pathway, adjusted for age and sex, estimating the indirect (a × b), direct and total effects with 95% percentile confidence intervals from 5000 bootstrap resamples (random seed = 42). For the phenotype comparisons displayed in the heatmap, Kruskal–Wallis *p*-values were corrected for multiple comparisons using the Benjamini–Hochberg false discovery rate, and pairwise differences were examined with Dunn’s test (Bonferroni-adjusted). Software: Python 3.11, statsmodels 0.14. Statistical significance was set at *p* < 0.05.

## 3. Results

The analytical sample included 2087 adults with a mean age of 62.6 ± 10.6 years. Women exhibited a higher prevalence of slow gait (77.1% vs. 60.5%; *p* < 0.001), slower gait speed (0.671 vs. 0.762 m/s; *p* < 0.001), and lower balance (7.96 vs. 8.55 s; *p* < 0.001). Men showed higher systolic (141.5 vs. 138.7 mmHg; *p* = 0.003) and diastolic blood pressure (80.4 vs. 78.4 mmHg; *p* < 0.001). PP, body mass index (BMI), HbA1c, handgrip strength, and vitamin D did not differ significantly by sex. Table 1 presents the complete characteristics stratified by sex.

**Figure 1:**
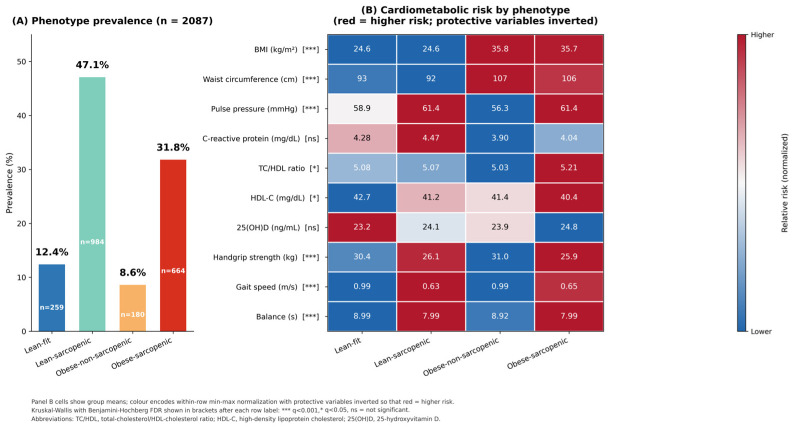
(**A**) Prevalence of the four phenotypes (*n* = 2087). (**B**) Heatmap of cardiometabolic risk by phenotype (red = higher risk; protective variables were inverted). Because multiple biomarkers and functional indicators are compared across the four phenotypes, *p*-values were corrected for multiple testing using the Benjamini–Hochberg false discovery rate (FDR); after correction, BMI, waist circumference, pulse pressure, handgrip strength, gait speed and balance remained significant (q < 0.001), with the total-cholesterol/HDL-C ratio and HDL-C at q < 0.05, whereas C-reactive protein (q = 0.79) and 25(OH)D (q = 0.24) did not; so, the heatmap is intended primarily for descriptive visualization. Abbreviations: PP, pulse pressure; CRP, C-reactive protein; HDL-C, high-density lipoprotein cholesterol; HbA1c, glycated hemoglobin; 25(OH)D, 25-hydroxyvitamin D; WHtR, waist-to-height ratio. Statistics: Kruskal–Wallis test (all variables non-normally distributed, Shapiro–Wilk *p* < 0.001).

**Figure 2:**
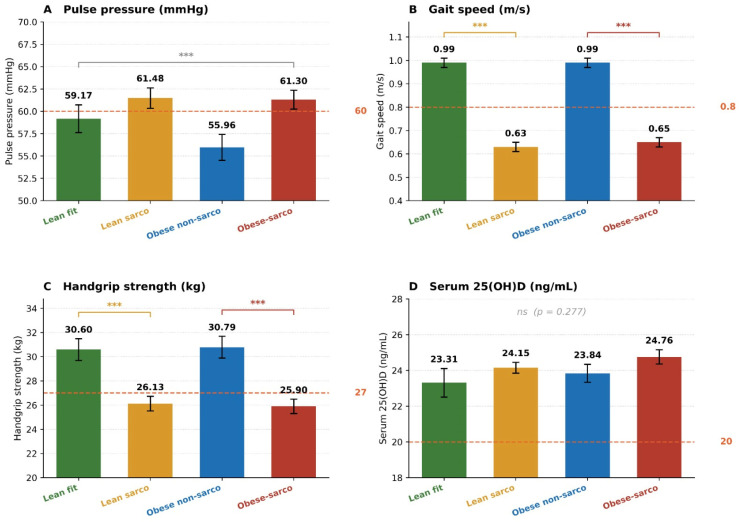
Obese–sarcopenic paradox by phenotype. (**A**) Pulse pressure. (**B**) Gait speed. (**C**) Handgrip strength. (**D**) Serum 25(OH)D. Bars represent means; error bars indicate SEM; dashed lines denote clinical thresholds. *** *p* < 0.001; ns = not significant (Kruskal–Wallis).

The lean–sarcopenic phenotype was the most prevalent (47.3%), whereas the obese–non-sarcopenic was the least common (8.7%). The distribution and cardiometabolic profile according to phenotype are shown in Figure 1 and Figure 2, with detailed values provided in Table 2.

The heatmap (Figure 1B) revealed that the obese–sarcopenic phenotype had the worst functional profile. Of note, and paradoxically, the obese–non-sarcopenic phenotype displayed the lowest PP (56.0 mmHg), suggesting that adiposity in the absence of muscle function loss does not promote arterial stiffening. The distribution of the four phenotypes and their detailed cardiometabolic profile are presented in Table 2 and Figure 1.

### 3.1. Strong-to-Weak Gradient by Handgrip Strength Tertiles

**Figure 3:**
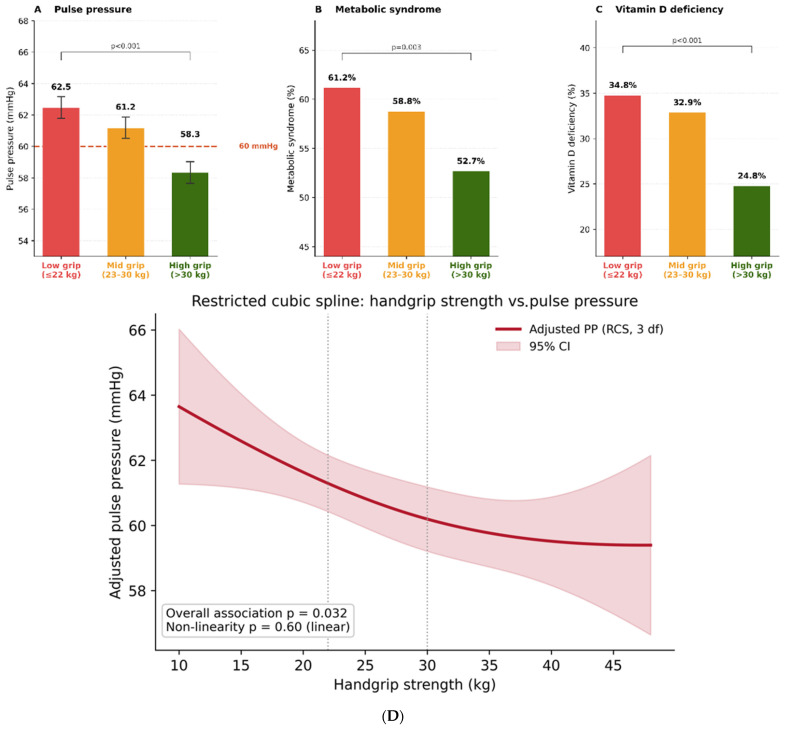
Strength gradient across handgrip strength tertiles. (**A**) Pulse pressure. (**B**) Metabolic syndrome prevalence. (**C**) Vitamin D deficiency prevalence. Of note, BMI remained similar across all tertiles (non-significant), indicating that the observed gradient is attributable to muscle function rather than adiposity. (**D**) Restricted cubic spline of continuous handgrip strength versus pulse pressure, adjusted for age and sex (natural cubic spline, 3 degrees of freedom; shaded band = 95% CI). The overall association was significant (*p* = 0.032), with no evidence of departure from linearity (test for non-linearity *p* = 0.60), supporting an approximately linear dose–response relationship.

**Figure 4:**
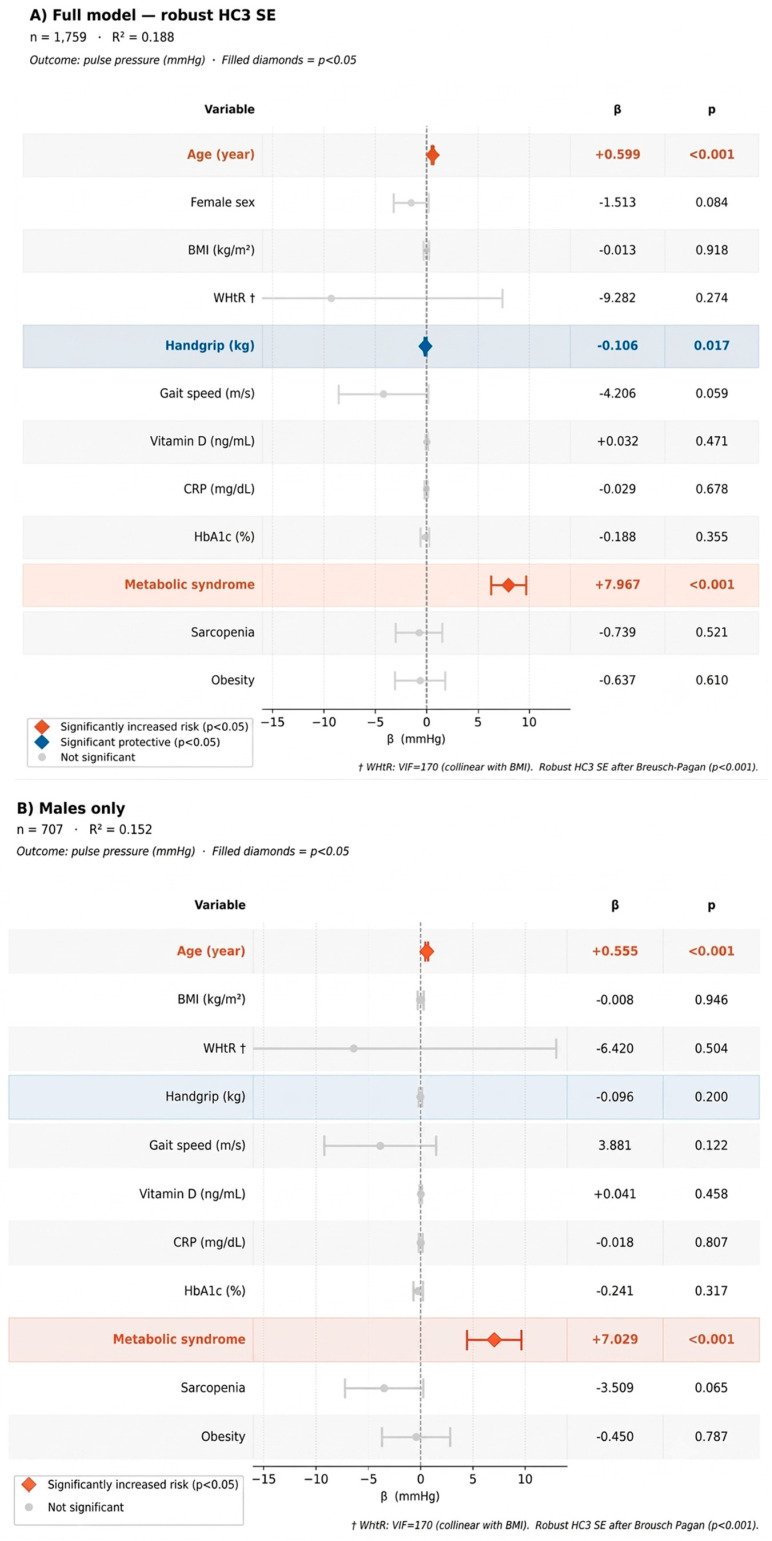

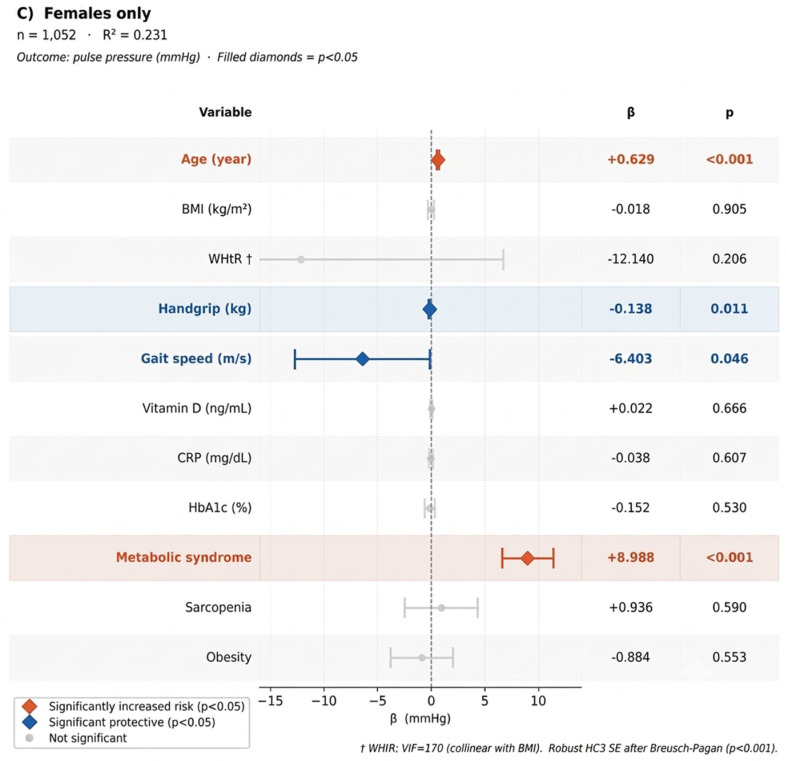
(**A**) Sex-adjusted OLS model corrected for multicollinearity (*n* = 1757; R^2^ = 0.207). BMI was removed from the set of continuous predictors due to high collinearity with WHtR (r = 0.85; original VIF > 100); all VIF values in the final specification were < 5. HC3 robust standard errors were used to account for heteroscedasticity (Breusch–Pagan *p* < 0.001). Significant predictors: age, metabolic syndrome, WHtR, and handgrip strength. (**B**) Sex-stratified model—men only, corrected for multicollinearity (*n* = 706; R^2^ = 0.162). After removing BMI, metabolic syndrome (β = +6.06 mmHg; *p* < 0.001) and age (β = +0.59 per year; *p* < 0.001) were the only significant predictors in men. Handgrip strength was not significant (β = −0.05; *p* = 0.488). (**C**) Sex-stratified model—women only, corrected for multicollinearity (*n* = 1051; R^2^ = 0.248). After removing BMI, handgrip strength (β = −0.13 per kg; *p* = 0.010) and gait speed (β = −6.38 m/s; *p* = 0.048) emerged as independent predictors exclusively in women; WHtR reached marginal significance (β = −13.86; *p* = 0.057). Metabolic syndrome (β = +8.06; *p* < 0.001) and age (β = +0.64 per year; *p* < 0.001) remained the dominant predictors.

The dose–response relationship was confirmed when analyzing the handgrip strength tertiles (Figure 3). This linear dose–response pattern was formally corroborated by restricted cubic spline modeling of continuous handgrip strength adjusted for age and sex: the overall association with PP was significant (*p* = 0.032), with no evidence of departure from linearity (test for non-linearity *p* = 0.60), supporting a graded approximately linear relationship rather than a threshold effect (Figure 3D). Across increasing strength tertiles (from lowest to highest), PP decreased progressively from 62.5 to 61.2 to 58.3 mmHg (*p* < 0.001, Kruskal–Wallis); metabolic syndrome prevalence declined from 61.2% to 58.8% to 52.7% (*p* = 0.003, Kruskal–Wallis); and vitamin D deficiency prevalence fell from 34.8% to 32.9% to 24.8% (*p* < 0.001, Kruskal–Wallis). Of note, the BMI remained similar across tertiles (29.0, 29.2, and 29.4 kg/m^2^, respectively; non-significant). Because the BMI was similar across tertiles, this stepwise gradient is unlikely to be explained by differences in general adiposity; consistent with this, the protective handgrip–PP association persisted after full adjustment for adiposity in the multivariable model (β = −0.09 per kg; *p* = 0.033; Figure 4A). A non-significant BMI difference does not, however, exclude residual confounding by adiposity, and no causal “driving” role can be inferred from this cross-sectional design; the gradient is therefore best described as an independent association of muscle strength with PP.

### 3.2. Multivariable Models, Diagnostics and Sex-Adjusted Results

In the robust OLS model adjusted for sex, after removing BMI from the continuous predictors to resolve collinearity with WHtR (*n* = 1757; R^2^ = 0.207; all VIF < 5), metabolic syndrome was the dominant predictor of PP (β = +7.20 mmHg; 95% CI: 5.62–8.78; *p* < 0.001), followed by age (β = +0.62 per year; 95% CI: 0.55–0.70; *p* < 0.001). WHtR, once freed from collinearity with BMI, emerged as an independent predictor of central adiposity (β = −13.34 per unit WHtR; 95% CI: −24.65 to −2.02; *p* = 0.021); the negative sign reflects residual confounding due to simultaneous adjustment for dichotomous obesity and gait speed, and is addressed in the Limitations. Handgrip strength remained an independent protective predictor (β = −0.09 per kg; 95% CI: −0.17 to −0.01; *p* = 0.033). (Figure 4A). CRP, HbA1c, sarcopenia, and dichotomous obesity did not reach significance after mutual adjustment. The magnitude and direction of the core effects (metabolic syndrome, age, handgrip strength) were robust to specification. Notably, the WHtR coefficient changed from non-significant in the original collinear model to significant in the corrected specification, confirming that variance inflation had masked the independent effect of central adiposity.

### 3.3. Age-Stratified Trajectories

**Figure 5:**
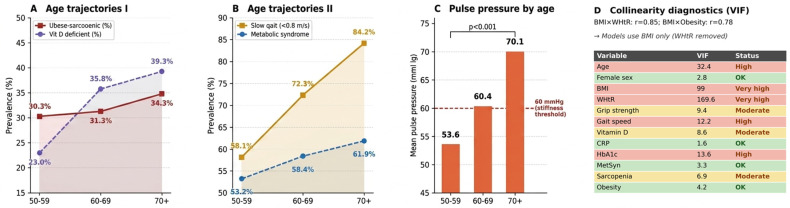
Age-stratified trajectories. (**A**) Obese–sarcopenic phenotype and vitamin D deficiency. (**B**) Slow gait and metabolic syndrome. (**C**) Mean pulse pressure (60 mmHg threshold indicated). (**D**) Variance inflation factor (VIF) collinearity table: green, VIF < 5; yellow, VIF 5–10; red, VIF > 10.

The obese–sarcopenic phenotype followed a modest but consistent age-related gradient (30.3% → 31.3% → 34.8% in the 50–59, 60–69, and ≥70 years age groups, respectively), whereas vitamin D deficiency increased more rapidly (23.0% → 35.8% → 39.3%). PP crossed the 60 mmHg threshold between the 50–59 and 60–69 years strata, defining a key intervention window. Figure 5 includes the VIF diagnostic table.

## 4. Discussion

The most important structural finding of this study is the unprecedented magnitude of the sex disparity in metabolic syndrome: 69.3% in women versus 40.1% in men, a difference of 29.2 percentage points that substantially exceeds the 77.4% overall prevalence reported in Mexican adults aged ≥ 60 years (71.2% in men, 83.7% in women) by González-Rocha et al. using ENSANUT 2016 data [15]. This disparity is consistent with the high frequency of metabolic syndrome reported in Mexican women (51.2%) by the National Survey of Cardiovascular Risk Factors, particularly among postmenopausal women, who exhibit a nearly three-fold higher odds of presenting multiple risk factors (OR 2.91, 95% CI 2.56–3.31) [27]. This disparity, combined with a higher prevalence of slow gait (77.1% vs. 60.5%) and slower gait speed (0.671 vs. 0.762 m/s) in women, configures a syndemic in which neither obesity nor arterial stiffness serve as the common denominator, as the PP and BMI were statistically similar between sexes (60.3 vs. 61.1 mmHg for PP; non-significant), but rather metabolic and functional dysfunction. The near-identical PP between sexes rules out arterial stiffness as the sole driver of the metabolic difference.

Regarding the first objective, the phenotype distribution revealed that lean–sarcopenic individuals constituted the largest group (47.3%), while only 12.3% of older Mexican adults were lean–fit. This high prevalence of sarcopenia in non-obese individuals suggests that muscle function deterioration is not exclusively driven by obesity but may reflect age-related loss independent of adiposity, consistent with the early onset of sarcopenic muscle loss documented in Mexico [13]. The fact that only 8.7% were obese–non-sarcopenic indicates that obesity rarely occurs without concurrent functional impairment in this population. Regarding the second objective, our findings support that handgrip strength is an independent protective predictor of the association between central adiposity (waist circumference) and arterial stiffness (pulse pressure), consistent with an independent protective association rather than a formal mediating role (formally tested below). The dose–response gradient across grip strength tertiles, combined with the independent protective effect of grip strength in the multivariable models (β = −0.09 per kg; *p* = 0.033), satisfies the associational preconditions for mediation (Baron-Kenny approach): (i) adiposity (WHtR) is associated with PP, (ii) adiposity is associated with grip strength (lower WHtR correlates with higher grip strength), and (iii) grip strength remains a significant predictor of PP after adjusting for adiposity. To move beyond these associational preconditions, we performed a formal mediation analysis (bootstrapped indirect effect, 5000 resamples) of the waist circumference → handgrip strength → PP pathway, adjusted for age and sex. The indirect effect was small and non-significant (indirect effect = −0.001; 95% CI −0.005 to 0.003 using waist circumference, and 0.018; 95% CI −0.035 to 0.083 using waist-to-height ratio), and the total effect of adiposity on PP was negligible. Handgrip strength therefore did not statistically mediate the adiposity–PP relationship; the data support an independent protective association of muscle strength with PP rather than a mediating pathway. Accordingly, mediation terminology has been removed from the study aims, results, and conclusions, and the relationship is described throughout as an independent association.

Postmenopausal estrogen withdrawal simultaneously accelerates visceral adiposity, insulin resistance, and endothelial dysfunction in Mexican women aged 50–80 years [15,28]. This biological transition overlaps with reduced physical activity, amplifying biological risk. National data from Mexico corroborate this trend, showing that physical inactivity is particularly high among older adults (25.1%) and has declined more steeply in women aged 50–59 years than in other age groups over the past two decades [29]. Longitudinal evidence further indicates that women are 75% more likely than men to remain persistently inactive across their life course [30], while the post-COVID-19 pandemic era has seen sedentary time increase significantly among postmenopausal women [31]. The net result documented here empirically for the first time in a nationally representative cohort is an excess of metabolic syndrome in women that more than doubles that in men and remains invisible to body weight-based screening.

The strength gradient confirms that each additional kilogram of handgrip strength is associated with 0.09 mmHg lower PP (95% CI: −0.17 to −0.01; *p* = 0.033) in the fully adjusted model corrected for multicollinearity, independent of central adiposity. Sex-stratified analysis revealed that this effect is clinically relevant only in women (β = −0.13 per kg; 95% CI: −0.22 to −0.03; *p* = 0.010). In men, handgrip strength did not reach significance (β = −0.05; *p* = 0.488), with metabolic syndrome and age being the dominant predictors in both sexes. The sex-stratified models are shown in Figure 4B (men) and Figure 4C (women). This interaction suggests that skeletal muscle exerts vasculoprotective effects mediated by estrogen, which are lost after menopause. The proposed mechanism involves muscle-derived vasoprotective myokines (i.e., irisin) and insulin-stimulated glucose uptake. In postmenopausal women, estrogen loss reduces muscle irisin synthesis, vitamin D receptor (VDR) sensitivity, and endothelial insulin signaling. These mechanistic interpretations are speculative and hypothesis-generating; the cross-sectional design does not permit causal or mechanistic inference. Resistance training interventions might confer differential and significantly better vascular benefits in this group [32].

Vitamin D deficiency did not differ across the four phenotypes (Kruskal–Wallis *p* = 0.277) but showed a significant gradient across handgrip strength tertiles (34.8% vs. 24.8%; *p* < 0.001). This indicates that functional muscle impairment rather than the adiposity–sarcopenia category per se determines vitamin D status, likely through a self-reinforcing cycle: low handgrip strength → reduced outdoor activity → decreased cutaneous vitamin D synthesis → further muscle deterioration via VDR deficiency. However, because vitamin D did not differ across phenotypes after false-discovery-rate correction (q = 0.24) and was not independently associated with PP in the multivariable model, this pathway is reported descriptively only and is not emphasized as a principal finding; the proposed muscle–vitamin D cycle remains a hypothesis rather than an established mechanism.

The prevalence of SO in our cohort (31.7%) substantially exceeds estimates from European and Asian cohorts. This high prevalence is concerning given that SO is associated with a 95% higher risk of cardiovascular disease and a 64% higher risk of cardiovascular mortality [6]. For example, Scott et al. reported that only 0.3% of older men met the EWGSOP2 criteria for SO, while 9.6% met the less stringent ESPEN-EASO definition [33]. This discrepancy aligns with the notion that muscle strength is a more important predictor of cardiometabolic risk than muscle mass per se [10], and that low muscle mass alone is directly associated with arterial stiffness [11,12]. The sex disparity in metabolic syndrome (29.2 percentage points) surpasses that reported in NHANES (~10 percentage point difference in adults aged >50 years) and in the 2012 ENSANUT survey, where the prevalence of metabolic syndrome in Mexican adults aged ≥60 years was 77.4% [15], indicating that cardiovascular risk in Mexican women is exceptionally elevated. This disparity is further exacerbated by the fact that menopause is independently associated with increased arterial stiffness, even after adjusting for traditional cardiovascular risk factors [16]. 

The handgrip strength–pulse pressure relationship in women (β = −0.13 per kg) extends the findings of previous studies to a Latin American cohort with sex stratification. Leong et al. demonstrated in the Prospective Urban Rural Epidemiology (PURE) study that lower grip strength is a robust predictor of cardiovascular mortality and major cardiovascular events across 17 countries [34]. More recently, König et al. reported that each 5 kg decrease in handgrip strength is associated with a 0.08 m/s increase in aortic pulse wave velocity, directly linking low muscle strength to higher arterial stiffness in an older European cohort [35]. In the specific context of postmenopausal women, Park and Park found that lower handgrip strength is independently associated with higher 10-year cardiovascular disease risk using data from the Korea National Health and Nutrition Examination Survey [36]. Taken together, these findings support our observation that muscular strength exerts a vasculoprotective effect that is particularly relevant in women, and our study provides the first evidence of this association in a nationally representative Mexican cohort.

Our findings reveal a paradox that challenges conventional assumptions about obesity and cardiovascular risk. Although the obese–sarcopenic phenotype exhibited the worst functional profile, the obese–non-sarcopenic phenotype (characterized by obesity without loss of muscle function) showed the lowest PP (56.0 mmHg) among all four phenotypes, even lower than the lean–fit reference group. This observation suggests that adiposity, in the absence of concomitant muscle function deterioration, does not necessarily promote arterial stiffening. In other words, preserved muscle strength may confer vascular resilience despite excess body fat. This paradox aligns with the concept proposed by Stenholm et al., where overweight and obese individuals with high handgrip strength had lower mortality than normal-weight individuals with high strength [9]. From a mechanistic perspective, metabolically healthy obesity, a phenotype where insulin sensitivity and inflammatory profiles remain normal despite high BMI, may explain part of this paradox [37]. However, our data indicate that muscle function, not merely metabolic health, is the key discriminating factor. The clinical implication is that screening for SO should prioritize functional assessment (grip strength and gait speed) over anthropometric indices alone, since normal-weight individuals with poor muscle function (lean–sarcopenic) had higher PP than their obese–fit counterparts.

### Limitations

This study has several limitations. The cross-sectional design precludes causal inference. Sarcopenia was defined using EWGSOP2 functional criteria without DXA or bioimpedance assessment of direct muscle mass. Because muscle mass was unavailable, sarcopenia was operationalized as probable sarcopenia (low handgrip strength and/or slow gait speed); consequently, the prevalence of sarcopenic obesity reported here (31.7%) is likely overestimated relative to consensus definitions that require confirmed low muscle mass and should not be directly compared with studies using such definitions. Pulse pressure served only as a surrogate (proxy) marker of aortic stiffness and is not equivalent to reference methods such as carotid–femoral pulse wave velocity; so, the vascular interpretations are necessarily indirect, and no strong conclusions about arterial structural change can be drawn from PP alone. Moreover, several well-established confounders of the muscle–vascular relationship were not available among the analyzed MHAS variables and could not be modeled—notably use of antihypertensive, glucose-lowering and lipid-lowering medications, physical activity, smoking and alcohol consumption, dietary patterns, socioeconomic status, menopausal status, renal function, and prevalent cardiovascular disease; residual confounding from these factors cannot be excluded and may have biased the reported associations. The high correlation between BMI and WHtR (r = 0.85) precluded including both indices simultaneously as continuous predictors; we opted to retain WHtR (a better predictor of central risk) and exclude BMI from the continuous predictor block, while retaining the BMI for dichotomous obesity classification. Consequently, the reported coefficients for WHtR capture the combined effect of general and central adiposity, not their separate contributions. A sensitivity analysis reversing the selection (retaining BMI and excluding WHtR) did not alter the magnitude or significance of the core predictors (metabolic syndrome, age, handgrip strength). HbA1c was measured using a point-of-care device without cross-calibration. The sample covers only four states, which may not capture the full regional heterogeneity of all 32 Mexican states, particularly the socioeconomic and dietary diversity of southern and rural communities. The absence of triglyceride measurements precluded application of the full IDF criteria; accordingly, metabolic syndrome was operationalized using a modified four-component definition (central obesity + any two of: HbA1c ≥5.7%, elevated blood pressure, reduced HDL-C). HbA1c ≥5.7% was used as a surrogate for impaired fasting glucose, consistent with ADA diagnostic thresholds. The direction of potential misclassification bias is uncertain: excluding triglycerides may underestimate the prevalence if hypertriglyceridemia is common in this population, but using HbA1c (a more stable marker) may capture cases missed by single fasting glucose measurements.

## 5. Conclusions

In this nationally representative sample of older Mexican adults, SO, arterial stiffness, metabolic syndrome, and vitamin D deficiency converge in a syndemic that disproportionately affects women. The sex disparity in metabolic syndrome prevalence (i.e., 69.3% in women vs. 40.1% in men) represents the most urgent public health finding of this study. Handgrip strength was an independent protective predictor of arterial stiffness (β = −0.09 per kg; 95% CI: −0.17 to −0.01; *p* = 0.033), independent of BMI, with a clinically significant effect observed exclusively in women (β = −0.13; *p* = 0.010), underscoring the need for resistance exercise programs with sex-sensitive design. Furthermore, this protective association followed a dose-response gradient across grip strength tertiles, highlighting sex-specific pathways in sarcopenic obesity. Because this is a cross-sectional analysis, these findings identify associations rather than causal effects; handgrip strength is best interpreted as a candidate modifiable marker whose therapeutic potential requires confirmation in longitudinal and interventional studies.

BMI has dominated decades of preventive health policy in Mexico. The MHAS 2012 data demonstrate that by measuring only weight and height, the healthcare system is misclassifying high-risk individuals while failing to detect millions of older women with metabolic syndrome, slow gait, and progressive muscle loss. The time has come to consider incorporating the handgrip dynamometer and the three-meter walk test into primary care consultations for adults aged 50 years and older. These findings unveil the sarcopenic obesity paradox: adiposity without muscle function loss does not stiffen arteries, whereas poor muscle function confers cardiovascular risk even in normal-weight individuals.

## Figures and Tables

**Table 1:** Baseline characteristics stratified by sex (*n* = 2087).

	Total (*n* = 2087)	Women (*n* = 1248)	Men (*n* = 839)	*p*-Value ^†^
Age (years)	62.6 ± 10.6	61.9 ± 10.8	63.6 ± 10.1	<0.001
Urban residency (≥100,000 population), (%)	57.6%	60.2%	53.7%	0.004
BMI (kg/m^2^)	29.2 ± 7.0	29.1 ± 7.0	29.3 ± 7.0	0.519
Waist circumference (cm)	98.1 ± 12.5	97.8 ± 12.5	98.4 ± 12.6	0.311
Waist-to-height ratio (ICT)	0.635 ± 0.089	0.634 ± 0.089	0.636 ± 0.088	0.724
WHtR ≥ 0.5 (%)	93.2%	92.9%	93.7%	0.530
Systolic blood pressure (mmHg)	139.9 ± 21.8	138.7 ± 21.9	141.5 ± 21.6	0.003
Diastolic blood pressure (mmHg)	79.2 ± 11.7	78.4 ± 11.3	80.4 ± 12.0	<0.001
Pulse pressure: PP (mmHg)	60.7 ± 17.9	60.3 ± 18.3	61.1 ± 17.2	0.182
PP ≥ 60 mmHg (%)	46.5%	45.3%	48.2%	0.203
HbA1c (%)	6.91 ± 1.93	6.85 ± 1.86	7.01 ± 2.03	0.518
HbA1c ≥ 6.5%: diabetes diagnosis (%)	35.3%	34.3%	36.8%	0.269
Hemoglobin (g/dL)	14.74 ± 2.08	14.77 ± 2.06	14.70 ± 2.12	0.918
PCR (mg/dL)	4.26 ± 7.22	4.43 ± 8.11	4.01 ± 5.64	0.182
HDL-C (mg/dL)	41.2 ± 10.4	41.5 ± 10.4	40.6 ± 10.3	0.058
Cholesterol total (mg/dL)	200.4 ± 46.3	202.5 ± 48.2	197.3 ± 43.2	0.040
TSH (μUI/mL)	2.88 ± 5.57	2.79 ± 4.93	3.02 ± 6.40	0.437
Serum 25(OH)D (ng/mL)	24.2 ± 8.6	24.2 ± 8.8	24.3 ± 8.4	0.637
Vitamin D deficiency < 20 ng/mL (%)	30.8%	31.4%	30.0%	0.510
Metabolic syndrome (%) ^†^	57.6%	69.3%	40.1%	<0.001
Handgrip strength (kg)	27.0 ± 9.1	26.9 ± 9.2	27.1 ± 8.9	0.609
Gait speed (m/s)	0.708 ± 0.236	0.671 ± 0.227	0.762 ± 0.239	<0.001
Slow gait < 0.8 m/s (%)	70.4%	77.1%	60.5%	<0.001
Maximum balance (s)	8.20 ± 2.95	7.96 ± 3.05	8.55 ± 2.76	<0.001
Probable sarcopenia (%) ^‡^	79.0%	79.1%	78.9%	0.952
Obese–sarcopenic phenotype (%)	31.7%	31.2%	32.3%	0.634

Values are presented as mean ± SD for continuous variables and percentages for dichotomous variables. Comparisons were performed using the Mann–Whitney U test (continuous variables) and the chi-square test (dichotomous variables). ^†^ Sex difference for metabolic syndrome: *p* < 0.001 (women 69.3% vs. men 40.1%). ^‡^ Probable sarcopenia: low handgrip strength and/or gait speed < 0.8 m/s (EWGSOP2 criteria). Values in red indicate *p* < 0.05.

**Table 2:** Cardiometabolic profile by phenotype (sex-adjusted in multivariable models).

	Lean–Fit	Lean–Sarcopenic	Obese–Non Sarcopenic	Obese–Sarcopenic	*p*-Value *
*n* (%)	257 (12.3%)	987 (47.3%)	182 (8.7%)	661 (31.7%)	
BMI (kg/m^2^)	24.6 ± 3.5	24.7 ± 3.7	35.8 ± 5.7	35.7 ± 5.0	<0.001
Waist circumference (cm)	92.8 ± 10.3	92.0 ± 10.1	107.3 ± 12.5	106.4 ± 10.1	<0.001
PP (mmHg)	59.2 ± 16.4	61.5 ± 18.2	56.0 ± 17.9	61.3 ± 17.8	<0.001
CRP (mg/dL)	4.28 ± 5.8	4.46 ± 8.9	3.91 ± 4.8	4.05 ± 5.2	0.790
HbA1c (%)	6.84 ± 2.0	6.98 ± 2.0	6.87 ± 1.8	6.86 ± 1.9	0.065
HDL-C (mg/dL)	42.6 ± 11.4	41.3 ± 10.0	41.5 ± 10.7	40.4 ± 10.4	0.039
25(OH)D (ng/mL)	23.3 ± 8.2	24.2 ± 8.2	23.8 ± 7.4	24.8 ± 9.6	0.220
Gait speed (m/s)	0.99 ± 0.15	0.63 ± 0.20	0.99 ± 0.14	0.65 ± 0.20	<0.001
Handgrip strength (kg)	30.6 ± 8.0	26.1 ± 9.3	30.8 ± 7.5	25.9 ± 9.0	<0.001
Maximum balance (s)	9.01 ± 2.4	7.99 ± 3.1	8.90 ± 2.3	7.99 ± 3.1	<0.001
TC/HDL ratio	5.08 ± 2.2	5.07 ± 1.6	5.03 ± 1.5	5.22 ± 1.5	0.039
Metabolic syndrome (%)	47.1%	50.9%	64.8%	69.6%	<0.001

Values are presented as mean ± SD or percentage. PP = pulse pressure; CRP = C-reactive protein; TC/HDL = total cholesterol/HDL cholesterol ratio. * Kruskal–Wallis test for comparisons across phenotypes. For PP, Dunn’s post hoc test (Bonferroni-adjusted) showed that the obese–non-sarcopenic group had significantly lower PP than the lean–sarcopenic (*p* = 0.001) and obese–sarcopenic (*p* < 0.001) groups, while it did not differ significantly from the lean–fit group (*p* = 0.33).

## Data Availability

The data presented in this study are openly available in the Mexican Health and Aging Study (MHAS) repository. The original data presented in the study are openly available at https://mhasweb.org/. Restrictions apply to the availability of the 2012 biomarker and physical fitness data used in this specific analysis, which were collected from a subsample of participants. These data are available from the corresponding author upon reasonable request and with the permission of the MHAS Steering Committee.

## Abbreviations

The following abbreviations are used in this manuscript:

ADA	American Diabetes Association
BMI	Body Mass Index
CI	Confidence Interval
CRP	C-reactive Protein
DBP	Diastolic Blood Pressure
DXA	Dual-energy X-ray Absorptiometry
ENSANUT	National Health and Nutrition Survey
ESPEN-EASO	European Society for Clinical Nutrition and Metabolism—European Association for the Study of Obesity
EWGSOP2	European Working Group on Sarcopenia in Older People 2
HbA1c	Glycated Hemoglobin
HC3	Heteroscedasticity-consistent Estimator Type 3
HDL-C	High-Density Lipoprotein Cholesterol
HR	Hazard Ratio
hs-CRP	High-sensitivity C-reactive Protein
IDF	International Diabetes Federation
INSP	National Institute of Public Health
MHAS	Mexican Health and Aging Study
NHANES	National Health and Nutrition Examination Survey
OLS	Ordinary Least Squares
OR	Odds Ratio
PCR	C-reactive protein
PP	Pulse Pressure
PURE	Prospective Urban Rural Epidemiology
SBP	Systolic Blood Pressure
SD	Standard Deviation
SEM	Standard Error of the Mean
SO	Sarcopenic Obesity
TC/HDL	Total Cholesterol/High-Density Lipoprotein ratio
TSH	Thyroid-stimulating Hormone
VDR	Vitamin D Receptor
VIF	Variance Inflation Factor
WHtR	Waist-to-Height Ratio
25(OH)D	25-hydroxyvitamin D
